# Maternal Satisfaction with Healthcare after Perinatal Loss in Monochorionic Twin Pregnancy

**DOI:** 10.3390/jcm8081213

**Published:** 2019-08-14

**Authors:** Mònica Druguet, Laura Nuño, Carlota Rodó, Silvia Arévalo, Elena Carreras, Juana Gómez-Benito

**Affiliations:** 1 Quantitative Psychology Unit, Faculty of Psychology, University of Barcelona, 08007 Barcelona, Spain; 2 Clinical Institute of Neuroscience (ICN), Hospital Clinic, 08036 Barcelona, Spain; 3 Maternal-Fetal Medicine Unit, Department of Obstetrics, Hospital Universitari Vall d’Hebron, Universitat Autònoma de Barcelona, 08035 Barcelona, Spain; 4 Group on Measurement Invariance and Analysis of Change (GEIMAC), Institute of Neurosciences, University of Barcelona, 08007 Barcelona, Spain

**Keywords:** maternal satisfaction, professional care, fetal surgery, perinatal loss, grief, monochorionic pregnancy, psychological difficulties

## Abstract

Introduction: The analysis of patients’ satisfaction with healthcare is recognised as being useful in the evaluation of health outcomes and perceived quality of care. Little is known, however, about how the psychological status of women who experience perinatal complications may affect their perceived satisfaction with care. Methods: We assessed healthcare satisfaction in 52 women who had undergone intrauterine surgery during a complicated monochorionic twin pregnancy and examined the influence that fetal loss and sociodemographic, clinical, and psychological factors had on the degree of satisfaction. Data were gathered in an individual interview and through the administration of the Medical Patient Satisfaction Questionnaire, Beck Depression Inventory, and State–Trait Anxiety Inventory. Relationships between variables were analysed using a chi-square test, Spearman’s rho, Student’s t test, and the Mann–Whitney U test, in accordance with the metric nature of the variables and the assumptions fulfilled. Results: Age and level of education were not associated with the degree of healthcare satisfaction. Negative but non-significant correlations were observed between the level of satisfaction and symptoms of anxiety and depression. Satisfaction with healthcare was high in the sample as a whole, although it was significantly higher among women who had not experienced fetal loss. There were no differences in satisfaction with services involving direct contact with medical staff, whereas satisfaction with indirect services was lower among women who had experienced perinatal loss. Conclusions: Due to the unique characteristics of this population, specialised care teams of both professional healthcare and indirect services are needed. Although administrative aspects of healthcare are regarded as being of secondary importance, this may not be the case with more vulnerable populations.

## 1. Introduction

Monochorionic twins account for 20%–25% of all twins. Monochorionic gestations have a higher risk of adverse outcomes due to the shared circulation between the twins. The placental anastomosis that connects the circulation of both twins can lead to particular complications such as twin-twin transfusion syndrome (TTTS), which can lead to the death of one or both fetuses if untreated. These complications affect one in five monochorionic pregnancies. For TTTS, intrauterine surgery can enable at least one fetus to survive in 80%–90% of cases, and both fetuses in 50%–75% of cases [1,2].

This situation is one of considerable psychological stress for the parents [3]. The severity of the complications and uncertainty over whether the fetuses will survive produces anxiety and may trigger anticipatory grief [4]. Furthermore, if one of the fetuses is ultimately lost, the associated grief has particular characteristics that can make it difficult to work through. The studies to date recognise that these parents have a complex set of emotional needs that may differ from those of parents who lose a singleton [5]. Pector and Smith-Levitin [6] suggest that despite the intensity of their grief for the deceased multiple, parents will often be denied “permission” from health professionals and family and friends to express this grief. Instead they will be encouraged to focus upon the “positives” of a surviving baby. A bereavement of this kind tends not to be recognised or expressed socially, and thus it is often dismissed, ignored, or underplayed [5,7]. In many cases, therefore, there is no funeral or other kind of farewell ritual that might help parents to mourn their loss [7,8]. Together, these aspects can heighten the emotional impact of the perinatal loss on the mother and may lead to a complicated grief reaction and/or the development of a psychological disorder [9]. Richards [5] suggests that grief can be delayed for months or years whilst the focus is upon caring for survivors. Other studies [6,10] draw attention to the fact that grieving for a loss can cause an inability to attach to the survivor(s).

A number of possible factors have been studied in relation to the complex phenomenon of complicated grief following perinatal loss. The evidence to date is inconclusive regarding the influence of factors such as not having children prior to the loss, a history of miscarriage, or the gestational age at which the fetus or fetuses are lost [7,8,11]. However, there is greater consensus over the protective role played by social and family support and the mother’s previous mental health [12].

The lack of social recognition and of an adequate understanding of the mother’s experience among immediate family and friends makes it difficult for her to draw upon these usual support networks. Under such circumstances, health professionals can play a key role in offering support to the grieving mother, especially in the early stages of bereavement following the perinatal loss. This supportive role, together with the complex nature of this kind of pregnancy and its associated risks and complications, means that the mother’s wellbeing will be greatly influenced by the care she receives throughout the process. Many mothers who have suffered perinatal loss in a twin pregnancy have symptoms of mental health issues. This population has unique needs for specific treatment and care. However, no care programs have been developed for these mothers and their partners [13].

Very little quantitative research has been employed to assess maternal satisfaction after perinatal loss in twin pregnancies [14]. The analysis of patients’ global satisfaction with healthcare is recognised as being a useful tool in the evaluation of health outcomes and perceived quality of care [15]. It is therefore important to examine satisfaction with care among women who have experienced perinatal loss and to identify the factors that may increase their level of satisfaction.

Studies in non-perinatal samples have reported that satisfaction with care depends on the nature of the illness suffered and the degree of recovery after treatment, it being lower in patients who experience more severe health problems and/or greater psychological difficulties [15,16]. However, these aspects have not been widely studied in the perinatal period, and especially not in relation to miscarriage and perinatal loss [14,17].

In their systematic review, Crow et al. [16] concluded that the evidence was inconclusive regarding the impact of sociodemographic factors on satisfaction with care. In the specific case of perinatal samples, no significant relationship has been found between the level of satisfaction with healthcare and personal variables such as age and level of education [15,17]. Moreover, little is known about how the psychological status of women who experience perinatal complications may affect their perceived satisfaction with care. Although several studies have considered the factors that influence the grief process in the context of perinatal loss [8,11,18], very few have examined satisfaction with hospital care [19], especially among women with a monochorionic twin pregnancy.

It is important to recognise that although grief, including feelings of anxiety and depression, is a common and normal response to the loss of a fetus during a multiple pregnancy, it usually diminishes after a year or so. However, the abovementioned factors and the emotionally complex nature of such a loss mean that the associated grief reaction often becomes complicated and chronic. If the symptoms of anxiety and depression persist, they may reach levels consistent with a psychological disorder [5]. In fact, around one-fifth of women who lose a fetus continue to present clinical-level symptoms twelve months later [20]. It is therefore important to understand the factors that may confer vulnerability to chronic psychological problems in such a situation.

The aim of the present study is to examine the influence of perinatal loss and of certain sociodemographic, clinical, and psychological factors on the degree of satisfaction with care among women who have undergone intrauterine surgery due to complications during a monochorionic twin pregnancy. The study hypotheses are as follows:

1. A higher level of satisfaction is associated with the survival of both babies compared with the loss of one or both twins.
2. The presence of psychological symptoms such as anxiety or depression is associated with lower satisfaction with care.
3. The level of satisfaction is associated with the number of weeks of gestation at delivery and the presence of complications in any surviving babies.
4. The degree of satisfaction is independent of the women’s age and level of education.

## 2. Materials and Methods

### 2.1. Design

This was a cross-sectional comparative correlational study.

### 2.2. Sample

Of the 82 women who fulfilled the inclusion criteria, 30 did not wish to participate in the study (participation rate of 63.4%). The sample therefore comprised 52 women who, in the course of a monochorionic twin pregnancy, underwent intrauterine surgery in the Maternal–Fetal Unit of the Vall d’Hebron University Hospital (Barcelona).

Of these women, 24 gave birth to two surviving babies, while the remaining 28 lost at least one of the fetuses. All the women were White and of Spanish nationality, and they had a level of education and command of the Spanish language that was sufficient for them to complete the questionnaires and respond to the interview questions. They all signed written informed consent forms, and the study was approved by the Ethics Committee of the Vall d’Hebron Hospital (ethical approval code ID-RTF066).

### 2.3. Measures

We prepared an interview schedule to gather sociodemographic information, including age, marital status, level of education, employment, status, life stressors, and the participant’s perception of family and social support (direct question with a yes/no answer). Data were also collected about personal and family psychiatric history and about each woman’s obstetric history. The final section of the interview asked about any complications and treatment undergone during the monochorionic twin pregnancy, fetal development, delivery, and the clinical follow-up of any surviving newborns.

In addition, the women completed the following three questionnaires:

#### 2.3.1. Medical Patient Satisfaction Questionnaire (MPSQ)

This 11-item questionnaire was developed to assess patients’ overall satisfaction with a variety of treatment aspects [21]. Items are answered using a five-point Likert scale ranging from one (strongly disagree) to five (strongly agree). The MPSQ has two subscales: items 1 to 6 evaluate satisfaction with direct contact with medical staff (for example, “My doctor’s care has helped me significantly”), while items 7 to 11 assess satisfaction with indirect services (for example, “I have easy access to my doctor’s office”). The authors of the original questionnaire [21] gave permission for the MPSQ to be adapted for use in the present study. Analysis of internal consistency in our sample yielded Cronbach’s alphas of 0.923 for the total score, 0.971 for the direct contact subscale, and 0.848 for the indirect services subscale.

#### 2.3.2. Beck Depression Inventory (BDI)

The validated Spanish version of this instrument [22,23] was used to assess symptoms of depression. Scores on the BDI range from 0 to 63, and higher scores are indicative of more severe symptoms of depression. In the present study, the instrument had a Cronbach’s alpha of 0.87.

#### 2.3.3. State–Trait Anxiety Inventory (STAI)

We used the Spanish version of this instrument [24,25] to assess both state anxiety (a transitory emotional experience) and trait anxiety (a relatively enduring disposition to feelings of anxiety). Each of the instrument’s subscales (State and Trait) comprises 20 items that are answered using a four-point Likert-type scale ranging from zero (“not at all’” on State subscale, “almost never” on Trait subscale) to three (“very much so” on the State subscale, “almost always” on the Trait subscale). Total scores for both state and trait anxiety therefore range from 0 to 60, with higher scores indicating greater anxiety. In the present sample, Cronbach’s alpha for the State and Trait subscales was 0.85 and 0.88, respectively.

### 2.4. Procedure

These 52 women were contacted by telephone between one and three years after fetal therapy, and were invited to participate in the study. Those who accepted were asked to give verbal informed consent before the collection of sociodemographic information, psychiatric history, and clinical data regarding their pregnancies.

In addition to these interviews, all participating women were sent copies of the three questionnaires (MPSQ, BDI, and STAI). Once they had completed the questionnaires, they sent them back to the hospital by email or in a prepaid envelope, together with a signed informed consent form.

### 2.5. Data Analysis

Quantitative variables were described as mean, standard deviation, median, minimum, and maximum. Categorical variables were described as frequencies and percentages. Prior to testing the hypotheses, we checked the normality of variables using the Kolmogorov–Smirnov test (*n* ≥ 30) and the Shapiro–Wilk test (*n* < 30), and also ensured that the corresponding assumptions were fulfilled for all the statistical tests used.

The relationship between categorical variables was studied by means of the chi-square test or, in the event that the condition for its application (E ≥ 5) was not satisfied, by Fisher’s exact test. In order to examine the relationship between quantitative variables we calculated Spearman’s rho correlation coefficients. The relationship between quantitative and categorical variables was studied by means of the Student’s t test and the non-parametric Mann–Whitney U test. The non-parametric Wilcoxon test for related samples was used to study the difference between quantitative variables. The level of significance for all tests was set at α = 0.05.

All analyses were performed using SPSS v19. The data collected can be consulted in the Supplementary Material.

## 3. Results

The sample comprised 52 women. In 24 cases (46.2%; mean age, 36 years) fetal surgery enabled both babies to survive, with a mean of 32.3 weeks of gestation at delivery. Among the 28 women who experienced perinatal loss (mean age, 35.7), one of the twins survived in 78.8% of cases (22 women), with a mean of 34.5 weeks of gestation at delivery. In the group of women with two surviving twins, complications were present in 79.2% of cases, and 76.3% of these babies required NICU (neonatal intensive care unit) admission. In the perinatal loss group, 36% of surviving babies presented complications, and of these, 11.1% were admitted to the ICU. Table 1 details the rest of the sociodemographic and clinical data for both groups.

Regarding the cause of death in the fetal loss cases, 55.9% were due to umbilical cord occlusion (44.1% following an intervention for selective fetal growth restriction and 11.8% after intervention for malformation) and 29.4% due to surgical complications associated with laser coagulation therapy. In the remaining 14.7% of cases, death occurred after the mother gave birth due to complications associated with an immature (prior to 24 weeks: 5.9% of cases) or premature birth (24+ weeks: 8.8% of cases).

The overall results from the MPSQ showed that women who did not experience perinatal loss were significantly more satisfied with the care they received (*p* = 0.03), thus confirming our first study hypothesis. A more detailed analysis of the instrument’s two subscales revealed that the difference concerned the level of satisfaction with indirect services (*p* = 0.02), since the degree of satisfaction with direct medical care was similar in the two groups (*p* = 0.58). In the sample as a whole, satisfaction with direct medical care was significantly higher than that regarding indirect services (*p* < 0.01).

The level of satisfaction with both direct care and indirect services showed a trend towards an inverse association with symptoms of anxiety and depression, with correlations ranging between –0.11 and –0.26 (see Table 2 for correlations by each subscale). However, none of these associations were statistically significant, neither for the sample as a whole nor for either subgroup. The results do not, therefore, support our second study hypothesis.

Finally, the level of satisfaction was not associated with sociodemographic (age and education) or clinical variables (number of weeks of gestation at delivery and the presence of complications in any surviving babies) in either of the two groups of women (Spearman’s rho and Mann–Whitney U with *p* > 0.05). These results support our fourth study hypothesis and refute the third.

## 4. Discussion

The results of this study show that women who have not experienced perinatal loss report higher overall levels of satisfaction, and that this was particularly the case for indirect aspects of healthcare (e.g., ease of making appointments, friendliness of administrative staff) compared to women who have suffered perinatal loss. The pregnancy outcome (i.e., perinatal loss vs. the survival of both twins) was not, however, a significant factor when it came to the level of satisfaction with direct medical care (based on indicators such as wishing to be treated by the same medical team in the future or recommending them to friends and relatives).

It should be noted that in the sample as a whole the majority of women were satisfied with the medical care received, and significantly more so than they were with indirect services. In fact, it was with respect to the latter that we observed differences between women who experienced perinatal loss and those who did not. In women whose pregnancy is marked by severe complications, the process needs to be closely monitored and managed. The medical teams who work in this area and who are in direct contact with expectant mothers have high levels of specialist training, making it more likely that they will treat women in a professional way and with effective emotional support. It is essential for health professionals to be actively aware of and recognise women’s varying emotional needs to grieve following a pregnancy loss, and to have the confidence to deliver sensitive, compassionate patient support as required. This would account for the positive response of the women in our sample.

By contrast, less attention tends to be paid to the quality of more administrative aspects of care, such as appointment making or relationships with administrative staff. These aspects nonetheless contribute to the overall care received and, therefore, they may influence patients’ perceived satisfaction. The fact that the women in our sample who lost one or both fetuses were significantly less satisfied with the indirect care they received highlights the importance of taking these aspects into account. For example, when women needed to contact health professionals, hospital procedures were also frequently identified as problematic, with the difficulty of arranging a visit or checking with the doctor.

In line with previous research [8], and as we hypothesised, the women’s age and level of education were not associated with the degree of healthcare satisfaction. As regards the impact of psychological variables, negative but non-significant correlations were observed between the level of satisfaction and symptoms of anxiety and depression. This suggests that while there may be an association between these variables, the relationship is not strong enough to yield statistical significance, at least not in a sample of the size employed here.

The results of the review by Crow et al. [16] suggest that satisfaction is linked to prior satisfaction with healthcare, the respondent’s predisposition, service utilisation, and the granting of patients’ desires (e.g., for tests). It is also affected by health status and health outcomes. Specifically, sicker patients and those experiencing psychological distress tend to report lower satisfaction. These conclusions, together with our findings here, underline the importance of ensuring that health professionals are sufficiently trained to offer support to mothers who have experienced perinatal loss. This support must be consistent with their emotional state, which implies communicating information in ways they are able to take on board and providing them with resources to help them cope with what is a devastating experience [7,26].

The small sample size, which was due to the low prevalence of the phenomenon being considered, was the main limitation of this study and might have compromised the power thereof. Due to the small number of participants, no modelling was performed to account for potential confounding factors or moderators/mediators. A further limitation is that we relied on convenience sampling and women’s retrospective accounts of their loss. A final limitation to consider was the high percentage of eligible women who declined participation. Although all the characteristics that might potentially distinguish this group of women from those we included could not be studied, we did verify that there were no differences between the two groups in terms of age, the type of complication experienced during pregnancy or the number of fetal losses.

Given that after perinatal loss, grief (including feelings of anxiety and depression) is a common and normal response, which diminishes after a year or so [20], this study focused on the relationship of maternal satisfaction with healthcare once the grief was no longer in the acute phase and could no longer mediate the results. Future studies could examine the relationship of such loss with the concurrent maternal satisfaction at the time of loss.

While acknowledging these limitations, we believe that the study provides valuable information about a delicate and little-studied problem. In fact, its main strength is precisely that it examines healthcare satisfaction in a highly specific population, namely monochorionic twin pregnancies involving perinatal complications. Our study also has important clinical implications. Women who experience perinatal loss are less satisfied with indirect attention services. Training programs for professionals and hospital staff who work with women who experience fetal loss could be a first step toward acknowledging the effect of perinatal loss and ensuring that mothers receive proper care. Healthcare professionals should therefore aim to validate and give meaning to the mother’s experience of loss, offering support and helping her to express her pain [7]. It is important to recognise different coping styles and grief patterns. It is recommended that care be taken to respect the individual wishes and needs of the mother as the standardisation of bereavement care runs the risk of disrupting a mother’s unique style of coping [14].

## 5. Conclusions

The loss of a fetus during a multiple pregnancy is a relatively uncommon event, the unique characteristics of which make it a singular experience for the mother, and it is therefore crucial to recognise that these women are more vulnerable to psychological problems [27]. Care of this kind can serve not only to reduce these women’s personal distress but also to achieve healthcare satisfaction.

In conclusion, overall satisfaction in this study was lower among women who experienced perinatal loss, but their level of satisfaction with direct medical care was comparable to that of women who did not lose either of the twin fetuses. The fact that the loss of a baby in such circumstances is often not acknowledged socially, and that many of these women do not receive the support they need from friends and family, highlights the key role of health professionals in such situations, a role that encompasses not only medical aspects but also the provision of adequate emotional support during the early stages of bereavement. This underlines the importance both of specialist training for professionals and of individualised care for these women. A crucial element here is the availability of stable and well-coordinated teams that are capable of building trust and a therapeutic alliance with patients, the ultimate goal being to improve both the actual and the perceived quality of care. A further point to consider is that although the more administrative aspects of healthcare are often regarded as being of secondary importance, this may not be the case with more vulnerable populations such as women who have experienced perinatal loss. Indeed, our results suggest that these administrative aspects should be regarded as a basic component in the provision of adequate and comprehensive care.

As noted by Britton [28], satisfaction is a multidimensional construct that may be influenced by many factors, and more research is clearly needed to explore how different types of illnesses and health outcomes may affect evaluations. In the specific case of mothers who have experienced a complicated monochorionic twin pregnancy, a more in-depth understanding is required of the sociodemographic, clinical, and psychological factors that may affect their satisfaction with healthcare, thus enabling the identification of those women who might benefit from more individualised care. Consistent with some of our findings, Geller et al. [19] concluded that the provider’s attitude can be an extremely important component in a woman’s satisfaction with pregnancy loss aftercare. In this respect, further research is also needed to explore the effect of different incentive structures on physician behaviours and patient satisfaction [16,29]. The ultimate goal of all this research should be to ensure that women have access to the information, social support, and professional care they need to help them deal with any mental health problems they may experience in the context of childbearing.

## Figures and Tables

**Table 1:** Descriptive data for sociodemographic, clinical, and psychological variables according to whether perinatal loss occurred.

	Perinatal Loss (*n* = 28)		No perinatal Loss (*n* = 24)	
	*n*	%	*n*	%
Marital status, married	28	100.0	22	91.7
Higher education	14	50.0	10	41.7
Social/family support, yes	18	53.6	19	79.2
Catholic faith	25	89.3	20	83.3
Previous children, yes	7	25.0	7	29.2
History of miscarriage	12	42.9	6	25.0
Fertility treatment	0	0	4	16.7
History of psychological problems	12	42.9	8	33.3
Current psychological treatment	6	21.4	1	4.2

**Table 2:** Correlations (*p* values) between Medical Patient Satisfaction Questionnaire (MPSQ) scores and those obtained on the Beck Depression Inventory (BDI) and State–Trait Anxiety Inventory (STAI).

	BDI	STAI	STAI
		State	Trait
MPSQ-Total	–0.14 (0.35)	–0.19 (0.20)	–0.26 (0.08)
MPSQ-Direct contact	–0.11 (0.47)	–0.11 (0.45)	–0.16 (0.30)
MPSQ-Indirect services	–0.13 (0.37)	–0.19 (0.19)	–0.26 (0.08)

## Supplementary Materials

The following are available online at https://www.mdpi.com/2077-0383/8/8/1213/s1, data S1: DataFile.sav.
